# Pathological Findings in White-Beaked Dolphins (*Lagenorhynchus albirostris*) and Atlantic White-Sided Dolphins (*Lagenorhynchus acutus*) From the South-Eastern North Sea

**DOI:** 10.3389/fvets.2020.00262

**Published:** 2020-05-27

**Authors:** Luca Schick, Lonneke L. IJsseldijk, Miguel L. Grilo, Jan Lakemeyer, Kristina Lehnert, Peter Wohlsein, Christa Ewers, Ellen Prenger-Berninghoff, Wolfgang Baumgärtner, Andrea Gröne, Marja J. L. Kik, Ursula Siebert

**Affiliations:** ^1^Institute for Terrestrial and Aquatic Wildlife Research, University of Veterinary Medicine Hannover, Foundation, Buesum, Germany; ^2^Division of Pathology, Department of Biomolecular Health Sciences, Faculty of Veterinary Medicine, Utrecht University, Utrecht, Netherlands; ^3^CIISA – Centre for Interdisciplinary Research in Animal Health, University of Lisbon, Lisbon, Portugal; ^4^Department of Pathology, University of Veterinary Medicine Hannover, Foundation, Hanover, Germany; ^5^Institute of Hygiene and Infectious Diseases of Animals, Justus-Liebig-Universität Giessen, Giessen, Germany

**Keywords:** pathology, *Lagenorhynchus albirostris*, *Lagenorhynchus acutus*, North Sea, Germany, The Netherlands

## Abstract

In the North Sea, white-beaked dolphins (*Lagenorhynchus albirostris*) occur regularly and are the second most common cetacean in the area, while their close relative, the Atlantic white-sided dolphin (*Lagenorhynchus acutus*), prefers the deeper waters of the northern North Sea and adjacent Atlantic Ocean. Though strandings of both species have occurred regularly in the past three decades, they have decreased in the southern North Sea during the last years. Studies describing necropsy findings in stranded *Lagenorhynchus* spp. are, to date, still scarce, while information gained through post-mortem examinations may reveal valuable information about underlying causes of this decline, including age structure and the reproduction status. Therefore, we retrospectively assessed and compared the necropsy results from fresh *Lagenorhynchus* spp. stranded along the southeastern North Sea between 1990 and 2019. A full necropsy was performed on 24 white-beaked dolphins and three Atlantic white-sided dolphins from the German and Dutch coast. Samples of selected organs were taken for histopathological, bacteriological, mycological, parasitological and virological examinations. The most common post-mortem findings were emaciation, gastritis and pneumonia. Gastritis and ulceration of the stomach was often associated with an anisakid nematode infection. Pneumonia was most likely caused by bacterial infections. Encephalitis was observed in three animals and morbillivirus antigen was detected immunohistochemically in one case. Although the animal also showed pneumonic lesions, virus antigen was only found in the brain. Parasitic infections mainly affected the gastro-intestinal tract. Lungworm infections were only detected in two cases and no associations with pathological alterations were observed. *Stenurus* spp. were identified in two of three cases of parasitic infections of the ears. Twelve of the 26 white-beaked dolphins stranded in Germany were found between 1993 and 1994, but there was no evidence of epizootic disease events or mass strandings during the monitored period.

## Introduction

The white-beaked dolphin (*Lagenorhynchus albirostris*, WBD) inhabits the temperate and subarctic waters of the North Atlantic and mainly occurs in shelf and shallow coastal waters (1). Little is known about the behavior and ecology of this marine mammal (2). The age of sexual maturity varies between 7 and 12 years in males, and 6 and 10 years in females. Male size ranges between 230 and 255 cm, while females tend to be smaller; 232–238 cm (3). Calving season takes place in the summer, after 11 months of gestation, as calves have been sighted from June to September (1). The calf length at birth ranges from 110 to 120 cm (4). Stomach content analysis revealed that whiting (*Merlangius merlangus*), cod (*Gadus morhua*), gobies (*Gobidae*) (5) and haddock (*Melanogrammus aeglefinus*) (2) play an important role in the diet of the WBD.

In the North Sea, WBDs are the second most common cetacean after the harbor porpoise (*Phocoena phocoena*) (6, 7). Abundance estimates conducted in the last 20 years indicated a relatively stable population with estimates from large scale surveys in the North Sea and adjacent areas ranging from 23,716 individuals (95% CI: 13,440–41,851) in 1994 [(8), revised in Hammond et al. (9)], 37,689 animals (95% CI: 18,898–75,164) in 2005 [(10), revised in Hammond et al. (9)] to 36,287 individuals (95% CI: 18,694–61,869) in 2016 (9). WBDs migrate to the north-western North Sea during summer months (June to August) with the occurrence peaking in August (11). A large scale spatiotemporal analysis on stranded WBDs from 1991 to 2017 across the North Sea area revealed a decrease in overall stranding numbers along the North Sea coast. A higher density of WBD strandings in earlier years could particularly be observed for the southern North Sea coastline, while increases in the densities in the north western area were seen more recently, and therefore suggest a shift in the distribution of the animals (12). A possible explanation for the observed trend difference was believed to be a difference in prey distribution and availability as a result of climate change. However, post- mortem findings, including causes of death, were not included in the study of Ijsseldijk et al. (12) and any changes in the health status of individuals, including the presence of infectious diseases, were therefore not considered when drawing these conclusions.

The Atlantic white-sided dolphin (*Lagenorhynchus acutus*, AWSD) inhabits the North Atlantic, prefers slopes and deeper waters and is therefore mainly seen offshore (13). The size of the adult animals drawn from different mass stranding events ranged from 218 to 243 cm for females and 249–274 cm for males (14, 15). Calving season starts in early summer with sightings of calves mainly ranging from June to September (16).

The AWSD is sighted less often in waters of the North Sea. The species is known to be highly mobile and travels long distances (16). Although genetic studies have found a difference between animals of the North Sea and other areas of the North Atlantic, it is assumed that there is no resident population living in the North Sea (16, 17). Population surveys made in the North Sea and adjacent waters regularly record AWSD individuals (2,187 [95% CI: 0–6071] in 2016), confirming their frequent passage in the area (9).

The protection status of the WBD as well as the AWSD is evaluated as ‘least concern‘ due to its wide spread abundance (18, 19). As for other marine mammal species, the main threats include those associated with human interactions (namely bycatch, small-scale hunting and contamination with anthropogenic compounds) (18, 19). Climate change, particularly the rise of the water level and temperature, may also contribute to the decline of the two *Lagenorhynchus* species in the future (12, 20, 21).

In order to assess the health status of marine mammals, systematic pathological investigations on population levels are necessary. Although the WBD is the second most common cetacean species in the North Sea, the knowledge on their health status is still scarce. Previous pathological findings from stranded WBDs in the North Sea included morbillivirus infection in a sub-adult live stranded animal on the North Frisian coast of Germany (22), parasitic infections with helminths such as *Anisakis simplex* and *Pholeter gastrophilus* (23), a classic vertebral osteophytosis in an animal found in Danish waters (24) and third stage deforming spondylosis as well as deformation and sclerosis of the bony plate in museum material of an adult female (25). The latter seems to occur frequently in adult individuals of this species (26). Parasitic infections and associated gastritis with penetrating ulcers, septicemia, enteritis, *Brucella* antibodies (detected in one animal) have been detected in animals stranded in Denmark (21).

Also, in the Netherlands, a virus with Rhabdovirus morphology has been described in a WBD (27). Skin disorders, such as lesions of possible infectious or traumatic origin, were described in animals from the Irish coast (28). Additionally, bacterial pneumonia (e.g., caused by staphylococci) and an infection with *Erysipelothrix rhusiopathiae* caused the death of six female WBDs in Newfoundland, Canada (29).

The aim of this study is to provide a comprehensive overview of the pathological findings of 24 white-beaked dolphins and three Atlantic white-sided dolphins stranded along the North Sea coast of Schleswig-Holstein, Germany, between 1990 and 2019 and along the North Sea coast of the Netherlands between 2008 and 2019. These findings, as well as the causes of disease and most likely causes of death, will help to evaluate results of future studies about these species and contribute to the assessment of risk factors, disease outbreaks and future stranding events.

## Materials and Methods

### Materials From Germany

Between 1990 and 2019, a total number of 26 WBD and two AWSD strandings were recorded on the North Sea coast of Schleswig-Holstein, Germany. The animals were either found dead or were euthanized due to severe illness without possibility of rehabilitation. As part of an on-going marine mammal health monitoring program, an element of the existing stranding network in the state of Schleswig-Holstein, the carcasses were transported to the Institute for Terrestrial and Aquatic Wildlife Research, University of Veterinary Medicine Hannover, Foundation. Depending on their state of preservation, carcasses were stored at −20°C until necropsy or were examined immediately after admission (for very fresh specimens). Of the 26 WBDs stranded in Schleswig-Holstein in the stated time period, 15 were considered suitable to perform a full post-mortem examination and for histological, microbiological and immunohistochemical investigations. Both AWSDs were considered suitable for further investigations.

### Materials From the Netherlands

Stranding records on the Dutch coast of the North Sea are maintained by Naturalis Biodiversity Center, Leiden and available online at http://www.walvisstrandingen.nl (30). A total of sixteen WBDs stranded between 2008 and 2019, of which nine animals were transported to the Faculty of Veterinary Medicine, Utrecht University (UU) for necropsies. One AWSD stranded in 2008 and was additionally collected for post-mortem examination at UU, while three other AWSDs stranded between 2010 and 2015, were not examined further. Animals that stranded prior to 2008 were not collected for necropsy at UU and therefore not described or included in this study. Ten animals stranded in the Netherlands (9 WBDs and 1 AWSD) were subject to a full macroscopic and histological examination.

### Gross Examination

All individuals were measured and weighed, and sorted into two age classes – immature and mature – according to total length measured from the rostrum to the fluke notch (3, 12, 14). Sexual maturity was also evaluated based on testicular and ovarian weight, presence of milk in the mammary glands or presence of a fetus (3). In 14 animals from the German coast and two from the Netherlands, four to six teeth from the middle of the lower jaw were removed for age determination based on annual growth layer groups, as described by Lockyer (31).

The necropsy examinations were performed according to international standardized guidelines (32, 33). The nutritional status was determined based on the state of development of the epaxial musculature, the total body weight and blubber thickness measured dorsally, laterally and ventrally in three different locations (sternal, cranial and caudal to the dorsal fin, DE) or only cranial to the dorsal fin (NL). Table 1 gives an overview of the conducted analyses per case for which a full necropsy was performed.

**Table 1:** Overview of additional examinations conducted per case.

**Case ID reference**	**Species**	**Country of examination**	**Full necropsy**	**Histology**	**Bacteriology**	**Immunohistochemistry**
WBD3	WBD	DE	x	x		
WBD5	WBD	DE	x	x	x	
WBD6	WBD	DE	x	x	x	
WBD7	WBD	DE	x	x	x	x (Morbillivirus)
WBD8	WBD	DE	x	x	x	x (Morbillivirus; *Toxoplasma gondii*)
WBD12	WBD	DE	x		x	
WBD13	WBD	DE	x	x	x	
WBD14	WBD	DE	x			
WBD15	WBD	DE	x	x	x	
WBD20	WBD	DE	x			
WBD21	WBD	DE	x			
WBD22	WBD	DE	x			
WBD24	WBD	DE	x	x		
WBD25	WBD	DE	x			
WBD26	WBD	DE	x	x	x	
AWSD1	AWSD	DE	x	x	x	
AWSD2	AWSD	DE	x	x	x	x (Morbillivirus)
LA1	WBD	NL	x	x		
LA2	AWSD	NL	x	x		
LA3	WBD	NL	x	x		
LA4	WBD	NL	x	x		
LA5	WBD	NL	x	x		
LA6	WBD	NL	x	x		
LA7	WBD	NL	x	x		
LA9	WBD	NL	x	x		
LA10	WBD	NL	x	x	x	
LA11	WBD	NL	x	x	x	
			27	22	12	3

*Only the 27 animals are listed, where a full necropsy was performed. DE, Germany; NL, the Netherlands*.

### Histology

Samples for histological examination were collected from a range of tissues, varying on a case-to-case basis, for fresh animals, including: adrenal gland, aorta, blubber, brain, diaphragm, esophagus, eye, gonads, heart, intestine (small and large), kidneys, liver, lungs, lymph nodes (retropharyngeal, pulmonary and mesenteric), mammary gland, nasal sinus, pancreas, rete mirabile, skeletal muscle, skin, spinal cord, spleen, stomach (1st, 2nd, and 4th compartment), thymus, thyroid gland, tongue, tonsils, trachea, urinary bladder and from tissues displaying gross lesions. These samples were fixed in 10% phosphate-buffered formalin, embedded in paraffin, cut at 4 μm and stained with hematoxylin and eosin (H&E). Selected sections were stained with elastica van Gieson stain, a trichrome stain for connective tissue. Furthermore, special stains were applied when histological lesions were suggestive of particular agents: Ziehl-Neelsen, to detect mycobacteria, Giemsa, to highlight protozoal organisms, or the periodic acid-Schiff (PAS) reaction, to detect fungi in the tissue.

### Parasitology

Parasitic infections were assessed macroscopically and level of infections graded semiquantitatively as mild, moderate or severe (34) during necropsy. Associated lesions were recorded and parasites were collected in tap water and later (after 1 h) fixed in 70% ethanol. After clearing with lactophenol or glycerine, specimens were identified according to morphological characteristics (35, 36), using a stereomicroscope (Olympus SZ61 Stereo Microscope).

### Microbiology

Samples of intestine, kidney, lung, liver, mesenteric lymph node and spleen were submitted for routine bacteriological and mycological investigation. Bacteria and fungi were cultivated as described by Siebert et al. (37) and identified using standard morphological and biochemical methods. Since 2014, identification is supported by a matrix-assisted laser desorption/ionization time-of-flight-analysis with Microflex LT/SH instrument as per manufacturer [Bruker Daltonics Bremen, Germany; (38–40)]. Additionally, samples of blood, blowhole, central nervous system, ear, esophagus, larynx, oral cavity, penis, placenta, pulmonary lymph node, nasal sinus, retropharyngeal lymph node, salivary glands, skin, thymus and uterus were submitted for bacteriological and mycological investigation, when gross lesions were of suspected bacterial origin [as described in Siebert et al. (32)].

### Immunohistology

Four WBDs and one AWSD from German waters showed macro- and microscopical lesions in the lung and/ or brain resembling those of morbillivirus infection. Therefore, these organs were examined immunohistologically with an immunoperoxidase technique for the presence of morbillivirus antigen. Different monoclonal antibodies [PDTC4, produced by the Institute of Virology, University of Veterinary Medicine Hanover, see also: Harder et al. (41); MCA387, MCA1893, produced by Serotec, no longer available] were used as described previously (22, 42). In one WBD displaying encephalitis, sections of the brain were immunohistochemically stained following a protocol of an avidin-biotin complex staining method using a polyclonal antiserum against *Toxoplasma gondii* (1:100) (43).

## Results

### Sex and Age Distribution

Twenty-five WBDs (60%, 25/42) were female and nine (21%, 9/42) were male. In eight of the WBD, sex determination was not possible. Sixteen females (64%) were mature, eight (32%) were immature and in one animal age was not possible to estimate. Only one male animal (11%) was mature, while the other eight males (89%) were immature. One female was pregnant with a male fetus and another female, which stranded alive, was accompanied by a calf. All AWSDs were males, two being mature and the other immature. Table 2 gives an overview on sex and age distribution of all WBDs.

**Table 2:** Sex and age classes of all WBDs stranded in Germany between 1990 and 2019 and in the Netherlands between 2008 and 2019.

	**Mature**	**Immature**	**NPD^a^**	**Total**
Male	1	8	0	9
Female	16	8	1	25
NPD	1	0	7	8
Total	18	16	8	42

a *NPD, No determination possible*.

**Figure 1:**
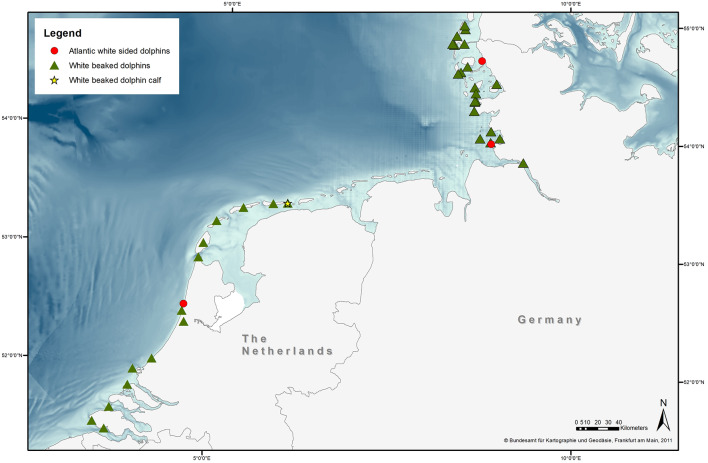
Stranding location of 40 white-beaked dolphins (triangles) and three Atlantic white-sided dolphins (circles) as well as one WBD calf (star) on the North Sea coast of Schleswig-Holstein and the Netherlands. Stranding location of one WBD from Germany was not possible to obtain. Stranding location of four WBDs and three AWSDs from the Netherlands is not pictured, as they only consisted of loose bones (Basemap provided by Bundesamt für Kartographie und Geodäsie, Frankfurt am Main, 2011).

Stranding locations are represented in Figure 1. The majority of strandings in Germany occurred between 1993 and 1994 (Recorded from 1990-2019, Figure 2). Most strandings (24%, 4/17) in The Netherlands occurred in 2011, but with an overall consistent distribution during the stranding period assessed in this study (Recorded from 2008 to 2019, Figure 2).

**Figure 2:**
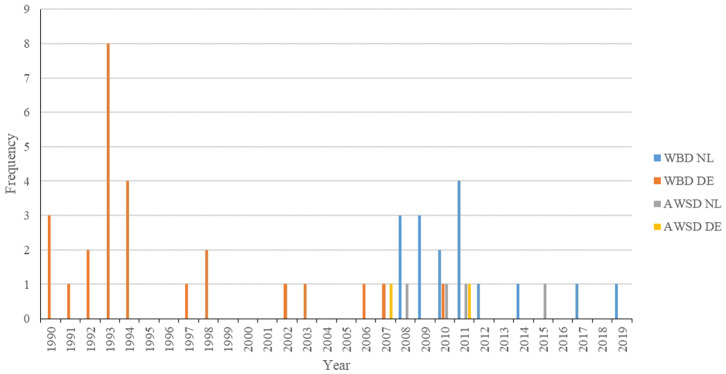
Year distribution of all strandings of white-beaked dolphins and Atlantic white-sided dolphins along the German and Dutch coast of the North Sea from 1990 to 2019. Strandings in the Netherlands prior to 2008 are not included, as they were not examined further at UU.

### Nutritional Condition

In general, the nutritional status of the studied animals was poor. Sixteen WBDs (38%, 16/42) were emaciated and eight (19%, 8/42) had a moderate nutritional status. Only two WBDs (5%, 2/42) were in a good nutritional condition. The nutritional status was not evaluated in 18 animals (43%, 18/42). All AWSDs were in a poor nutritional condition. The distribution of the nutritional status in relation to the age is shown in Table 3.

**Table 3:** Age classes of the white-beaked dolphins and Atlantic white-sided dolphins in relation to the nutritional status.

**Nutritional status**	**Age distribution**			
	**Mature**	**Immature**	**NPD^a^**	**Total**
Good	2	0	0	2
Moderate	6	2	0	8
Poor	8^*^	9^**^	0	17
Not evaluated	3	6	9	18
Total	19	17	9	45

a *No determination possible*.

* *Two Atlantic white-sided dolphins included*.

** *One Atlantic white-sided dolphin included*.

After a gross examination, 24 WBDs and the three AWSDs were considered suitable for further investigations, based on their state of preservation. In the following paragraphs only these 27 animals are taken into account and referred to by their case numbers (see Table 1). An overview of the macroscopic and histological findings, as well as the causes of death is given in Table 4.

**Table 4:** Overview of macroscopic and histologic findings of the 27 WBDs and AWSDs, that were fully examined during necropsy, and the causes of death.

**Case ID reference**	**Year of stranding**	**Sex**	**Age**	**Macroscopic**	**Histology**	**Cause of death**
WBD3	1998	F	M	Pleuritis; Stomach: gastritis (1st), parasites (all); Esophagus: Ulceration; Enteritis; Pyometra; Congestion of kidneys and liver	Lung: granulomatous pneumonia, suppurative bronchopneumonia; Stomach: ulcerative and (pyo)granulomatous gastritis; Brain: Lipofuscin accumulation; skin: Granulomatous-histiocytic dermatitis	Euthanasia
WBD5	2003	F	M	Stomach: gastritis (1st, 2nd), Parasites (1st, 2nd); Enteritis, intestinal parasites; Thymic cysts	Lung: Interstitial pneumonia, edema, emphysema; Stomach: Ulcerative, granulomatous/pyogranulomatous, hemorrhagic gastritis; Thymic cysts; Kidneys: atrophy of glomerula; Liver: lymphocytic hepatitis, periportal fibrosis	Endoparasitosis
WBD6	2006	F	M	Stomach: gastritis (1st, 2nd), Para sites (1st, 2nd); Enteritis, intestinal parasites; Otitis media; Skin: scars and nematodes in subcutis; Thymic cysts	Lung: suppurative bronchopneumonia, interstitial pneumonia, edema, emphysema, fibrosis, calcification; Stomach: eosinophilic, ulcerative gastritis, calcification; Skin: suppurative dermatitis and epidermal hyperplasia; Thymic cysts; Suppurative vaginitis and lymphohistiocytic inflammation of cervix; Kidneys: fibrosis; Liver: fibrosis	Cardiovascular failure due to live stranding
WBD7	2007	M	I	Stomach: gastritis (1st), Parasites (1st, 2nd); Skin: wounds, suppurative myositis	Lung: suppurative bronchopneumonia, interstitial pneumonia, emphysema; Stomach: granulomatous/pyogranuomatous gastritis; Brain: encephalitis, cell necrosis, calcification, malacia, astrogliosis, microgliosis, inclusion bodies; Skin: suppurative dermatitis/inflammation of the blubber	Euthanasia
WBD8	2010	F	I	Stomach: gastritis (1st, 2nd), Parasites (2nd)	Lung: suppurative bronchopneumonia, necro-suppurative pneumonia, edema, emphysema; Stomach: Ulcerative gastritis; Brain: necrotizing encephalitis	Euthanasia
WBD12	1991	M	I	Lung: edema; Stomach: Parasites (1st)		Unknown
WBD13	1992	F	I	Stomach: gastritis (2nd), parasites (1st, 2nd)	Lung: interstitial bronchopneumonia, edema, fibrosis; Stomach: Ulcerative gastritis	Unknown
WBD14	1992	F	M	Stomach: Ulceration (2nd) intestinal volvulus; Ears: nematodes liver: suppurative-granulomatous hepatitis, periportal fibrosis		Intestinal volvulus
WBD15	1993	F	M	Lung: edema; Stomach: gastritis (2nd), parasites (1st); Skin: suppurative inflammation of blubber, foreign body in skin; Skeletal system: ankylosis of vertebrae, ossification of humerus with radius/ ulna	Lung: interstitial pneumonia; Stomach: Granulomatous/pyogranulomatous gastritis	
WBD20	1993	M	I	Lung: parasites and parasitic nodules; Stomach: ulceration and parasites		Pneumonia
WBD21	1993	F	M	Stomach: ulceration (1st, 2nd), parasites (1st, 2nd); Intestine: Parasites; Esophagus: parasites		Emaciation
WBD22	1993	F	M	Lung: Parasitic nodules; Intestine: Parasites; Esophagus: Parasites		
WBD24	1994	M	I	Gastritis (1st)		Unknown
WBD25	1994	F	I	Gastritis (1st)		Unknown
WBD26	1994	F	M	Pleuritis; Stomach: Parasites (1st)	Lung: Interstitial pneumonia; Kidneys: Congestion; Liver: Congestion	Unknown
AWSD1	2011	M	M	Stomach: Parasites (1st); Intestine: Parasites; Ears and eustachian tube: Nematodes; Skin: irregular pigmentation, lesions; Subcutis: parasites	Lung: Edema; Stomach: Ulcerative gastritis (1st); Muscle, blubber: Calcification, lympho-histiocytic inflammation, parasites; Splenitis; Hepatitis	Splenitis, hepatitis
AWSD2	2007	M	I	Stomach: parasites (2nd); Ears: Nematodes; Subcutis: parasites, abscesses; Peritoneum: Parasites	Lung: Edema; Brain: encephalitis and meningitis	Encephalitis
LA1	2008	F	M	Stomach: Gastritis (1st), parasites (1st); Esophagus: Ulceration; Intestine: Parasites; Skeleton: Irregular new bone formation, sequestration, osteomyelitis; Subcutis: Hematoma	Lung: Pneumoconiosis; Kidneys: Necrosis of tubular epithelium; Liver: Necrosis with lymphoplasmacellular infiltrates, brown pigmentation of hepatic cells	Emaciation, trauma
LA2	2008	M	M	Lung: Edema, emphysema, congestion; Esophagus: Ulceration; Subcutis: Parasites, linear calcification		
LA3	2009	F	I	Lung: Edema; Stomach: Gastritis (1st, 2nd); Esophagus: ulceration	Stomach: necrosuppurative, ulcerative gastritis	Emaciation
LA4	2009	F	I	Ears: hematoma		Emaciation
LA5	2009	F	M	Lung: edema; Stomach: gastritis		
LA6	2010	F	I	Lung: edema	Lung: pneumoconiosis brain: hyperemia; Kidneys: necrosis of tubular epithelium liver: necrosis and lymphoplasmacellular infiltration, brown pigmentation of hepatic cells	
LA7	2010	M	I	Stomach: gastritis (1st)		Emaciation
LA9	2012	M	M	Stomach: parasites (all) thymic cysts; Spleen: swelling of red pulp	Lung: interstitial and pyogranulomatous bronchopneumonia, edema stomach: necrosuppurative gastritis (all); Brain: hyperemia, lipofuscin accumulation, satellitosis thymus: cysts, lymphoid depletion, necrosis; Lymphoplasmacytic cystitis; Liver: lymphohistiocytic hepatitis	
LA10	2017	M	M	Stomach: Gastritis (2nd), parasites (2nd); Intestine: Enteritis; Skin: Pox-like lesions; Throat: Abscess	Stomach: Granulomatous gastritis; Brain: Suspected encephalitis (hemorrhages, purulent nodules, necrosis of neurons, activated microglia), hyperemia, perivascular bleedings; Skin: swollen keratinocytes, inclusion body-like structures; Liver: periportal fibrosis	*Clostridium sordellii* septicemia
LA11	2019	F	M	Stomach: gastritis (2nd), parasites (2nd); Brain: hyperemia, perivascular bleeding, blood clot; Skin: white plaque on dorsal head, depressed scar on fluke, rake marks, pox-like lesions; Subcutis: Hemorrhages under white plaque and rake marks	Lung: edema, emphysema; Brain: Suspected encephalitis (hyperemia, hemorrhages, infiltration with eosinophils in cerebrum, bacterial colonies in cerebellum); Skin: Erosion and apoptosis (head), necrosis, accumulation of bacteria, eosinophils, neutrophils (fluke), ulceration and erosion (rake marks), erosion, apoptosis, hemorrhages (pox-like lesions); Spleen: Hyperplasia of white pulp; Kidneys: hyperemia, intersitial fibrosis, mineralization, cystic dilatation of tubular epithelium in right kidney; Liver: Hyperemia, vacuolization, roundcell and polymorph infiltration	Trauma

### Skin and Blubber

**Figure 3:**
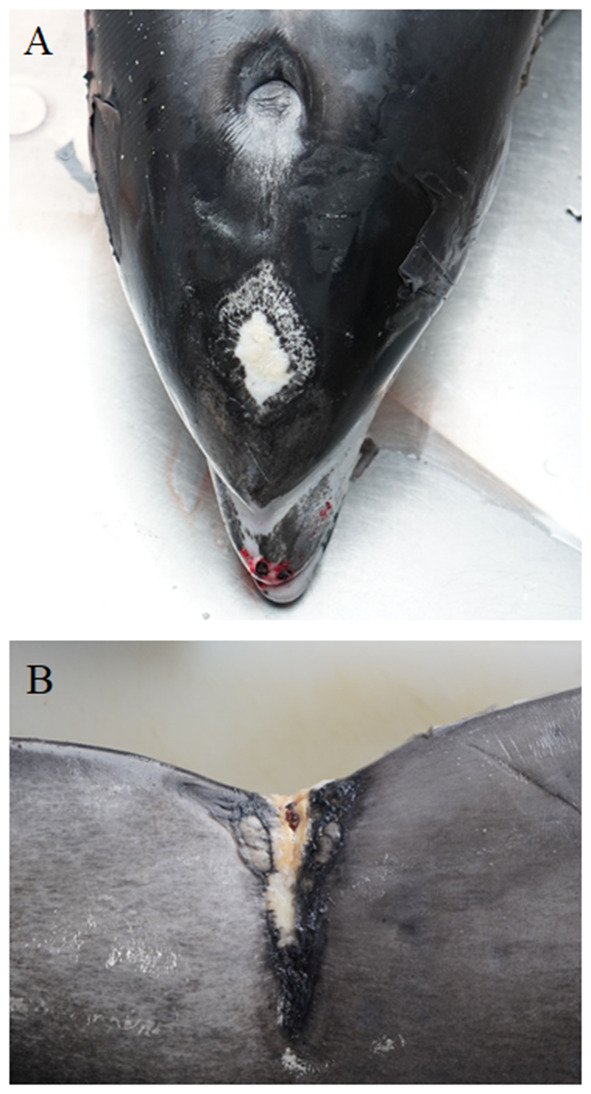
Elevated white plaque (~5 x 8 cm) cranial to the blowhole on the head of LA11 **(A)**. Depressed scar (~8 × 6 cm at its widest point) on the dorsal tailstock **(B)** of the same animal. (photo credit: Steve Geelhoed).

Granulomatous and histiocytic dermatitis was found in WBD3, which had a swelling on the upper lip. WBD6 had several older scars along one side of the body as well as a suppurative dermatitis with epidermal hyperplasia. In the fascia underneath the blubber of this animal, several nematodes were found, which were specified as *Crassicauda* spp. (Spiruromorpha: Tetrameridae). WBDs 7 and 15 showed suppurative inflammation of the blubber, but no parasite infection was detected in the skin/ sub cutis of these two animals. WBD7 also had superficial skin wounds and suppurative myositis. A microbiological examination revealed beta-hemolytic streptococci, *Edwardsiella tarda*, yeast, *Pseudomonas* spp., *Erwinia* spp. and *Enterobacter* spp. in WBD7. In WBD15 a foreign body (lower jaw of a horn pike) was perforating the skin and blubber on the right abdomen, ventral of the dorsal fin, and was therefore the cause of the inflammation. Origins of the foreign body of the skin lesion remained unclear and also if the skin lesions of the other animal were caused by trauma, inter- or intraspecific interaction or anthropogenic impact. LA11 showed several lesions of the skin and blubber. It had an elevated white plaque (Figure 3A) cranial to the blowhole on the rostrum with severe hemorrhage in the underlying tissue. The same animal also had multifocal hemorrhages in the brain, and it was assumed that blunt trauma to the head was the cause. Histology of these lesions showed erosions of the superficial epithelium, apoptotic epithelial cells and single granulocytes in the sub cutis. On the fluke, a large depressed scar was seen (Figure 3B), indicating a chronic traumatic lesion. The histological examination of the scar revealed severe ulceration, necrosis and accumulation of bacteria, eosinophils and neutrophils. On the lateral body flanks, several parallel lesions, including rake marks, with hemorrhage in the underlying tissue and microscopic ulcerations and erosions of the superficial layers were seen. Several dark, depressed patches on the lateral body resembling those of pox-virus infection were also examined microscopically. They showed erosion of the epithelium, hemorrhages and apoptosis, not typical for pox-virus infections. Pox-like lesions, found mainly on the head and tailstock, were detected on an additional WBD (LA10). Histologically, swollen keratinocytes and inclusion body-like structures could be seen in samples of the skin, but no definite histological identification for pox-virus could be made.

**Figure 4:**
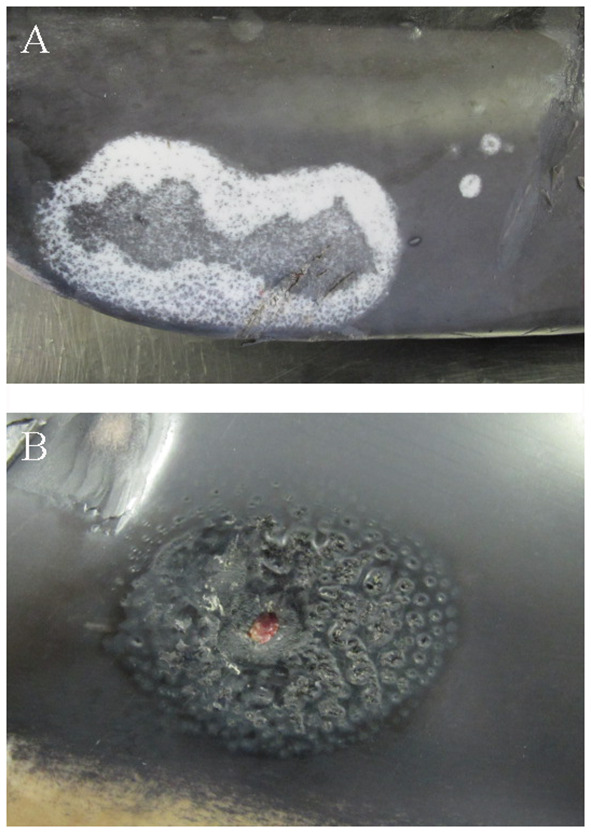
Skin lesions of AWSD1, showing severe discoloration on the right side of the abdomen, ~8 cm cranial to the fluke **(A)** and round shaped lesions with holes perforating into the blubber **(B)**.

**Figure 5:**
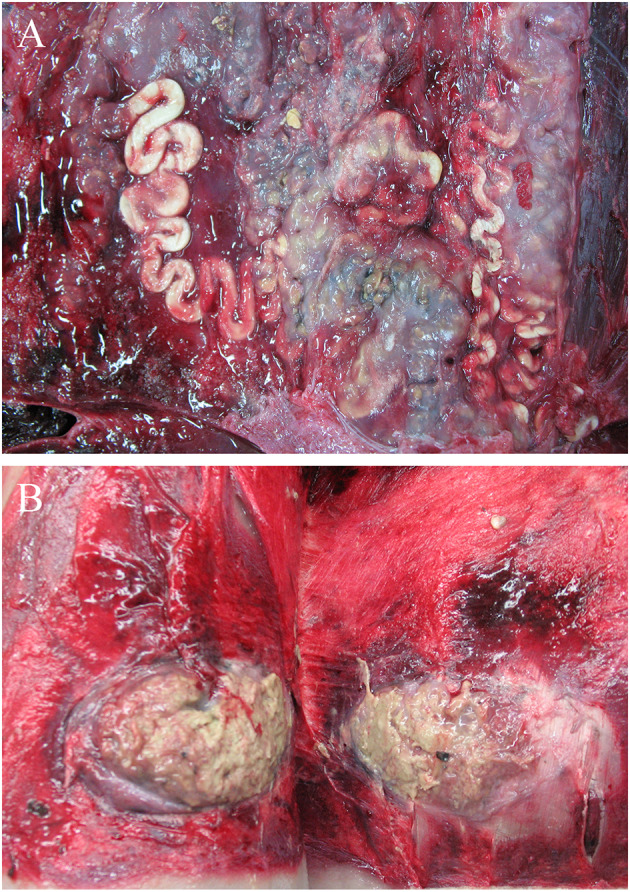
Parasite infection with nematodes (*Crassicauda* spp.) of the subcutaneous fascia in AWSD2 appeared on both sides of the caudo-lateral abdomen **(A)**. The same animal also displayed several abscesses in the musculature and sub cutis on the left side of the caudo-lateral abdomen, which were also infected by *Crassicauda* spp. **(B)**.

Two AWSDs (AWSD1, AWSD2) had multifocal alterations of the skin and subcutaneous tissue. AWSD1 had multiple irregularly pigmented areas of the skin of different sizes and shapes (1–17 cm) (Figure 4A) as well as round shaped lesions with multiple holes reaching into the blubber (Figure 4B). The skin lesions were examined microbiologically and gamma-hemolytic streptococci were detected. Additionally all investigated AWSDs (AWSD1, AWSD2, LA2) had infections with nematodes (*Crassicauda* spp.; Tetrameridae; Spirumorpha) along their dorso-lateral flanks. On both sides of the abdomen *Crassicauda* spp. infected the subcutaneous muscle and fascia of AWSD1. The histological examination showed multifocal, nodular calcification of the muscle and the blubber displayed multiple necrotic areas as well as lympho-histiocytic inflammation. In the musculature, a parasitic cyst was seen containing oval, 60–80 μm large parasite eggs. Similar to the first AWSD, *Crassicauda* nematodes (Tetrameridae; Spiruromorpha) infected the subcutaneous fascia of AWSD2 (Figure 5A). Several abscesses (up to 10 × 7 cm) were detected in the blubber and musculature of AWSD2 (Figure 5B). Histologically, the musculature and skin displayed granulomatous-necrotizing inflammation, parasites in the muscle and parasite eggs in muscle and skin. Linear calcifications in the blubber, resembling lesions of *Crassicauda* infection, were also seen in LA2, but the lesions were not as severe as in the other two animals.

AWSD1 and AWSD2 had infections with cestode larvae in the blubber, intra- and extraperitoneal fascia as well as musculature in different locations. Multiple parasitic cysts of varying size (0,7–3 cm in diameter) were seen upon gross examination of AWSD1. The cestode merocercoid morphotype *Phyllobothrium delphinii* (Phyllobotriidea) was isolated and identified from the blubber.

### Respiratory System

The major gross pathological finding was pneumonia, which was observed in nine WBDs (33%, 9/27) (WBD3, WBD5, WBD6, WBD7, WBD8, WBD13, WBD15, WBD26, LA9). Histologically, interstitial pneumonia (55%, 5/9) and interstitial bronchopneumonia (22%, 2/9), suppurative bronchopneumonia (44%, 4/9), granulomatous/ pyogranulomatous pneumonia (22%, 2/9) and necro-suppurative pneumonia (11%, 1/9) were detected. Additionally, two animals showed pleuritis (WBD3, WBD26), which is most likely associated with the lung inflammation, specified in WBD3 as suppurative and granulomatous and in WBD26 as interstitial. Microbiological examination was performed on six of the nine animals with pneumonia. Detected microorganisms included *Pseudomonas* spp. (*n* = 2), beta-hemolytic streptococci (*n* = 2), gamma-hemolytic streptococci (*n* = 2), coliform bacteria (*n* = 2), *Escherichia coli* (*n* = 2), yeast (*n* = 1), *Enterobacter* spp. (*n* = 1) and *Edwardsiella tarda* (*n* = 1) in cases with suppurative or interstitial pneumonia/ bronchopneumonia and *Erwinia* spp (*n* = 1), *Acinetobacter* spp. (*n* = 1), *Proteus* spp. (*n* = 1) and aerobic *Bacillus* spp. (*n* = 1) only in correlation with interstitial pneumonia/bronchopneumonia. Two animals (WBD7, WBD8) were selected and tested for morbillivirus antigen, as these two WBDs were also diagnosed with encephalitis in addition to the pneumonia. Antigen of morbillivirus was not detected in the lungs of these animals. A mild infection with lung nematodes was observed in two WBDs (WBD20, WBD22). The lung of WBD20 was mildly infected and displayed lungworm nodules macroscopically, as well as slightly enlarged lung lymphnodes. In WBD22, parasitic nodules were recorded on gross examination as well. In both animals, this was probably caused by pseudaliid nematodes (*Halocercus* spp.), but the lungs did not display further pathological alterations. Histopathological examinations were not conducted for these two WBDs. Other common pathological findings in the lungs of the examined WBDs included pulmonary edema (58%, 14/24) and emphysema (21%, 5/24). Similar to this observation, pulmonary edema was seen in all of the AWSDs (AWSD1, AWSD2, LA2) and emphysema and congestion in LA2.

Less common diagnoses included focal, interstitial, pulmonary fibrosis (WBD6, WBD13), pneumoconiosis (LA1, LA6) and multifocal pulmonary calcification in WBD6. WBD6 also showed calcification in the stomach wall. Hypercalcemia related to hyperparathyroidism could be suspected, as the animal also showed hyperplasia of the parathyroid, but no blood screening was performed to confirm this hypothesis.

### Gastrointestinal System

**Figure 6:**
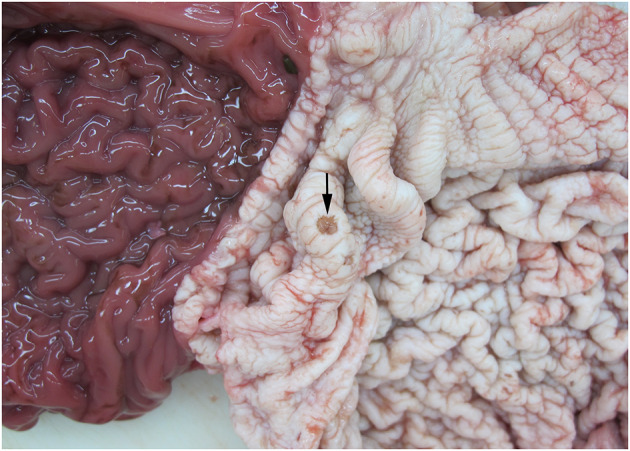
Gastric ulceration (arrow) in the 1st compartment of the stomach of WBD8.

Of the 27 examined animals (including the three AWSDs), 20 animals were diagnosed with gastritis (74%). In 12 animals (WBD3, WBD7, WBD13, WBD14, WBD15, WBD24, WBD25, AWSD1, LA1, LA7, LA10, LA11) the gastritis was restricted to one compartment of the stomach (first or second), five animals (WBD5, WBD6, WBD8, WBD21, LA3) had lesions in the first and second compartment and in LA9 all compartments were affected. For WBD20 and LA5, there was no further information on the location of the lesions. In six cases, gastritis was diagnosed solely macroscopically, with ulcers visible grossly ranging from a few mm to 6 cm in size (Figure 6). In 14 animals, pathohistological examination specified the gastritis as ulcerative (50%, 7/14), granulomatous/pyogranulomatous (36%, 5/14), necro-suppurative (14%, 2/14), eosinophilic (7%, 1/14) and/or hemorrhagic (7%, 1/14).

Eighteen animals (2 AWSDs, 16 WBDs) had parasitic infections of at least one stomach compartment. Parasites were detected in the lumen on gross examination (*n* = 10) and were seen histologically, penetrating the gastric wall in 8 cases. In 12 animals, parasites were identified as anisakid nematodes. LA10, from the Netherlands, was found to be infected with *Pseudoterranova decipiens* (Rhabditida: Anisakidae) in the 2nd stomach. AWSD1 and AWSD2 displayed an additional infection with the trematode *Pholeter gastrophilus* (Digenea: Heterophyidae) in the stomach wall (*ampulla duodenalis*). A combination of both gastritis and anisakid nematode infection was observed in 14 (82%, 14/17) animals. An overview of the parasitic infections in the different parts of the gastrointestinal tract is given in Table 5. Ulceration of the esophagus was detected in three WBDs (WBD3, LA1, LA3) and one AWSD (LA2). Three other WBDs (WBD6, WBD21, WBD22) had parasites in the esophagus, in WBD6 identified as anisakid nematodes. Enteritis was diagnosed in four cases (WBD3, WBD5, WBD6, LA10) and six animals presented parasitic infections of the intestine (WBD5, WBD6, WBD21, WBD22, AWSD1, LA1). In WBD6 the acanthocephalan *Bolbosoma capitatum* (Acanthocephala; Polymorphidae) was identified and associated with a pyogranulomatous enteritis. Acanthocephalan infections were recorded in two more cases (WBD21, WBD22). WBD5 showed a combination of parasitic infection and enteritis, but no identification of the parasites was performed. WBD14, a female, had an intestinal volvulus, associated with hemorrhagic infarction, severe bloating of the intestine and large amounts of dark red fluid in the abdomen. Apart from ulcerative gastritis, no other abnormalities of the gastrointestinal tract were observed in this animal.

**Table 5:** Distribution and level of parasitic infection found in the different compartments of the stomach and gastrointestinal system from WBDs from the German and Dutch North Sea.

	**Mild**	**Moderate**	**Severe**	**Without quantity**	**Total**
Esophagus	0	1	0	2	3
1st compartment	9	4	1	0	14
2nd compartment	3	5	2	2	12
3rd compartment	1	1	0	0	2
Ampulla duodeni^*^	0	2	0	0	2
Intestines^**^	2	0	0	4	6

*If not specified, the parasites were anisakid nematodes or not specified further*.

* *Pholeter gastrophilus*.

** *Bolbosoma capitatum, anisakid nematodes*.

### Central Nervous System

**Figure 7:**
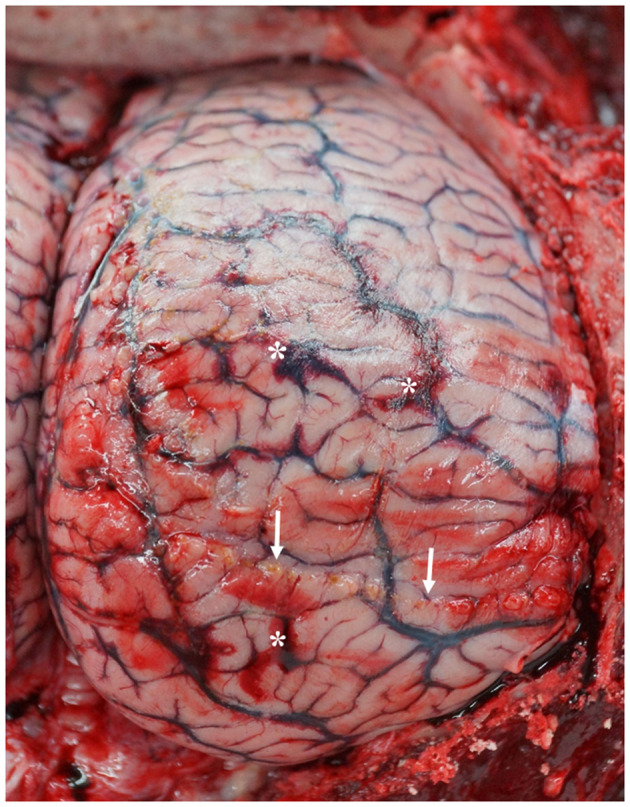
Dorsal view of the right hemisphere of the cerebrum with multifocal hemorrhages (stars) and purulent areas (arrows) in LA10 with acute meningoencephalitis. (photo credit: Steve Geelhoed).

Encephalitis was found in two WBDs (WBD7, WBD8) and one AWSD (AWSD2), an acute meningoencephalitis in one (LA10) and a mild meningitis in another (LA11). WBD7 had a non-suppurative encephalitis and also showed mild, perivascular lymphocytic infiltration, cell necrosis, moderate calcification, moderate, focal malacia, astrogliosis and microgliosis and moderate amounts of cytoplasmatic and nuclear, eosinophilic inclusion bodies in neurons and glial cells. WBD8 had a necrotizing encephalitis. AWSD2 had a non-suppurative meningitis in three localizations that were assessed, as well as non-suppurative encephalitis. LA10 and LA11 showed hemorrhages (Figure 7) and focal purulent nodules on the meninges as well as a larger amount of cerebrospinal fluid. The histological examination showed necrosis of individual neurons and activated microglia in LA10, while LA11 presented hyperemia and hemorrhages in several areas of the brain and infiltration with eosinophils in the cerebrum and bacterial colonies in the cerebellum. Other pathological findings of the central nervous system were hyperemia, seen in four WBDs (LA6, LA9, LA10, LA11), and mild perivascular hemorrhages in two cases (LA10, LA11). LA11, with severe hemorrhages and hyperemia, had a blood clot (3 × 1 cm) in the cerebellum, which may be indicative of a brain infarction, although other findings described for this animal in the skin and subcutis section also suggest a traumatic cause. No histological image of the specific area, where the blood-clot was located, was made to help determine the cause of this finding. Lipofuscin accumulation was detected in two WBDs (WBD3, LA9) and LA9 had a satellitosis in the cerebrum.

Immunohistochemical analysis was performed on the brain and lung tissue of WBD7, WBD8 and AWSD1, with the intent to detect a morbillivirus infection. Morbillivirus antigen was found in WBD7 and specified as dolphin morbillivirus (DMV). A detailed report on this case has been published previously (22).

Serology to detect antibodies against *Brucella abortus, Brucella melitensis* and *Leptospirae (L. canicola, L. grippotyphosa, L. tarassovi, L. icterohaemorrhagiae, L. pomona, L. hardjo, L. bratislava)* was performed for WBD7. The results were negative.

### Ears and Eyes

Three animals (WBD14, AWSD1, AWSD2) showed nematode infections in the tympanic bullae and cranial sinus. Nematodes were found in the Eustachian tube of AWSD1. Both AWSDs showed mild to moderate infections. Identification of the parasites revealed *Stenurus globicephalae* (Metastrongyloidea; Pseudaliidae) in AWSD1 and AWSD2. For WBD14 no speciation was performed. WBD6 had suppurative otitis media in both ears. No ear nematodes were detected in this animal. Microbiological examination detected *Salmonella* spp., *Pseudomonas* spp., *Klebsiella oxytoca* and *Clostridium perfringens*. LA4 showed dark red coloration of the subcutaneous tissue around the ears during gross examination, but no histological examination was performed. No abnormalities were detected in the eyes of any of the animals.

### Hematolymphatic System

Three WBDs (WBD5, WBD6, LA9), two mature females and an immature male, had multiple macroscopically visible cysts in the thymus, ranging from 1 to 2.5 cm in diameter. Histologically, the cysts of the female animals (WBD5, WBD6) were lined by epithelial cells and contained colloid in WBD6, while the male animal (LA9) showed lymphoid depletion and necrosis. Macroscopically, no other animal showed cysts of the thymus, but as histological examination of the thymus was only performed on eight animals, no definite statement can be made, especially concerning the occurrence of microcysts in WBDs.

One AWSD (AWSD1) had a splenitis. To detect certain pathogens, the tissue was stained with Giemsa, Ziehl-Neelsen and PAS, but all were negative. Bacteriological examination detected gamma-hemolytic streptococci. Apart from the splenitis, pathological findings in the spleen were rare. Macroscopic swelling of the red pulp (LA9) and histologically detected hyperplasia of the white pulp (LA11) were detected once in two WBDs, both together with extra medullary hematopoiesis.

### Skeletal System

Only two WBDs showed abnormalities of bone structures, both were mature females (WBD15, LA1). WBD15 was 5 years old by growth layers in the teeth and showed ankylosis of several vertebrae and ossification of the humerus with radius and ulna. In LA1, irregular new bone formation and sequestration of the left lateral spinous processes of the tail vertebrates and osteomyelitis was seen. The subepidermal tissue in the same area showed a large hematoma; trauma is likely to have caused the bone reaction.

### Urogenital Systems and Abdominal Cavity

A suppurative vaginitis with lympho-histiocytic inflammation of the cervix was found in WBD6. WBD3 had a moderate pyometra. No bacteriological, mycological or virological examination was performed on these animals.

Two WBDs (LA1, LA6) displayed a necrosis of the tubular epithelial cells of the kidneys and LA11 had hyperemic kidneys, interstitial fibrosis and mineralization of the renal parenchyma. Only the right kidney showed cystic dilatation of the tubules. Other pathological findings of the kidneys included congestion in two WBDs (WBD3, WBD26), atrophy of the glomeruli in one animal (WBD5) and one case of fibrosis (WBD6). In LA9 a lymphoplasmacytic cystitis was diagnosed. AWSD1 had a parasitic cyst in the peritoneum left of the urinary bladder close to the head of the epididymidis and several cysts in the mesenteries of the right testicle, which likely have a parasitic origin as well. Furthermore a 6 × 5 cm abscess with caseous content was located between the testicles.

In the peritoneal wall, close to the accessory glands, AWSD2 displayed parasitic cysts, containing cestode larvae of the morphotype *Monorygma grimaldii* (Diphyllobotriidea). The same animal also had a single nematode in the area of the papilla seminalis, suggestive of *Crassicauda* spp., which resembles the findings of nematodes in fascia and musculature of this animal (see above).

### Liver

Hepatitis was detected in three WBDs (WBD5, WBD14, LA9) and was specified macroscopically as suppurative-granulomatous with focal abscesses in WBD14 and histologically as multifocal, lymphocytic/lymphohistiocytic in WBD5 and LA9. Microbiological examination detected *Staphylococcus epidermidis, Pseudomonas* spp., *Erwinia* spp., *Acinetobacter* spp. as well as coliform bacteria in WBD5 and infection with *Clostridium sordellii* in LA10, without signs of hepatitis. No further tests were conducted for WBD14 and LA9. WBD14, with several lesions in the liver, also had an intestinal volvulus, mentioned in the “Gastrointestinal tract” section. AWSD1 had a multifocal, necrotizing hepatitis, together with focal complete vascular thrombosis. To detect certain pathogens, the tissue was stained with Giemsa, Ziehl-Neehlsen and PAS, but all were negative. No pathogens were detected during the bacteriological examination. The same animal also had a necrotizing splenitis, mentioned above, which was associated with gamma-hemolytic streptococci. Other pathological findings in the liver were periportal fibrosis (WBD5, WBD6, WBD14, LA10), congestion (WBD3, WBD26), multifocal necrosis with lymphoplasmacytic infiltration (LA1, LA6), cytoplasmatic, brown-yellow pigmentation in approximately 90% of the hepatic cells (LA1, LA6), hyperemia, vacuolization and periportal roundcell and polymorph infiltration (LA11).

### Immunohistochemistry

Immunohistochemical investigation of brain and lung samples failed to detect morbillivirus antigen in the studied animals from Germany, except for WBD7, which has been described previously (22). AWSD2 which was diagnosed with encephalitis was tested negative for morbillivirus. WBD8 was tested for the presence of *Toxoplasma gondii*, but results were negative. No immunohistochemistry was performed on the animals stranded in the Netherlands.

### Bacteriology and Mycology

Microbiological examination was conducted on 85 organ samples from 12 animals which were in a fresh state of conservation. Twenty nine bacterial species and yeasts were isolated. The most frequently detected bacteria, based on the number of organs they were detected in, were coliform bacteria *(26), Pseudomonas* spp. *(24)*, gamma-hemolytic streptococci *(18), E. coli (17)* and aerobic bacilli *(16)*. In association with pathological findings, *streptococci* (beta- and gamma-hemolytic species), *Pseudomonas* spp. and *coliform bacteria* were the most commonly detected microbes, mainly detected in the lungs, pulmonary lymph nodes and mouth/throat. The detection of multiple microorganisms in the same animal was observed regularly (*n* = 8). In Table 6 only bacteria and yeast are listed, that were detected in organs with macroscopic and/or histopathological lesions. Beta- and gamma-hemolytic streptococci, *E. coli*, coliform bacteria as well as *Pseudomonas* spp. were the most common microbes associated with pneumonia and were often found in the pulmonary lymph nodes as well. In WBD7, a serum analysis for antibodies against *Brucella abortus, Brucella melitensis* and *Leptospirae* as potential zoonotic agents was performed. All results were negative. *Clostridium sordellii* was cultured from the spleen, kidney, liver and tissue of an abscess in the musculature of the throat area of a WBD (LA10) diagnosed with septicemia. The kidneys, liver and spleen of the same animal did not present any pathological changes. A coinfection with *Streptococcus* spp. in the lung and *Edwardsiella tarda* in the area of the abscess was also observed.

**Table 6:** Microorganisms detected in organs with pathological lesions from WBDs and AWSDs stranded along the German and Dutch coast.

		**Number of organs with pathological findings and microorganisms**											
**Microorganisms**	**Cultured from *n* =**	**Liver**	**Lungs**	**Lung associated lymphnodes**	**Hearing apparatus**	**Thymus**	**Central nervous system**	**Mouth/ Throat**	**Spleen**	**Skin**	**Kidneys**	**Musculature/throat abscess**	**Case IDs of affected animals**
*Acinetobacter* spp.	12	1	1										WBD5
*Aerobic Bacilli*	16		1										WBD13
*Alcaligenes* spp.	3			1				1					WBD7
*Beta-hemolytic streptococci*	11		2	1		1		1					WBD6, WBD7, WBD26
*Clostridium perfringens*	9				1			1					WBD6, WBD7
*Clostridium sordellii*	4											1	LA10
*Clostridium* spp.	3		1						1		1		LA11
Coliforme bacteria	26	1	3	1				1	1		1		WBD5, WBD7, LA11
*Edwardsiella tarda*	7		1					1				1	WBD7, LA10
*Enterobacter* spp.	9		1	1				1					WBD7
*Erwinia* spp.	8	1	1					1					WBD5, WBD7
*Escherichia coli*	17		2	1									WBD6, WBD13
*Gamma-hemolytic streptococci*	18		2				1	1	1	1			WBD7, WBD8, AWSD1
*Klebsiella oxytoca*	1				1								WBD6
*Proteus* spp.	13		1										WBD13
*Pseudomonas* spp.	24	1	2	1	1			1					WBD5, WBD6, WBD7
*Salmonella enterica* subspp. (Group B)	1				1								WBD6
*Staphylococcus epidermidis*	5	1		1		1							WBD5, WBD6
Streptococci spp.	2		1						1				LA10, LA11
Yeast	15		1	1		1							WBD6, WBD7
Total	187	5	20	8	4	3	1	9	4	1	2	2	

## Discussion

The most common pathological findings diagnosed in white-beaked and Atlantic white-sided dolphins stranded along the South-eastern North Sea were emaciation, gastric ulceration, parasitosis and pneumonia. This resembles findings of a former study on *L. albirostris* strandings along the Danish coast of the North and Baltic Sea (21). Furthermore, the high incidence of parasitic infections of the skin, blubber and musculature in AWSDs is comparable with the results of a pathological study by Rogan et al. (15).

Gastritis and infection with anisakid nematodes were common and a correlation with anisakid nematode infections and gastric ulceration has been described (23, 32, 34). Parasitic infections were associated with gastritis in a majority of the animals (82%, 14/17). Although these lesions were severe in some cases, it was not considered the main cause of death of these animals. A contribution to emaciation has been assumed (21) and seems likely in this study, with the majority of animals with pathological findings of the stomach and/ -or parasitosis, regardless of the severity of the findings, being in a poor nutritional condition (54%, 13/24). Table 7 shows the relation between the nutritional status and pathological findings of the stomach. Emaciation was considered the cause of stranding or death in four animals from the Netherlands, but would have likely played a role in weakness and stranding of other dolphins investigated in this study as well.

**Table 7:** Nutritional condition in relation to the occurrence of pathological findings of the stomach (gastritis, parasitic infection) in WBDs and AWSDs from South-eastern North Sea.

	**Good**	**Moderate**	**Poor**	**Not evaluated**	**Total**
Parasitic infection	0	0	4^*^	0	4
Gastritis	1	1	2	2	6
Parasitic infection and gastritis	1	5	7^*^	0	13
No pathological findings	0	2	2^*^	0	4
	2	8	15	2	27

* *One AWSD included*.

For most stranded WBDs and AWSDs the ultimate cause of death could not be assigned and for those, in which a cause was determined (*n* = 12), this significantly varied between individuals (see Table 4). Emaciation was the most likely associated with spontaneous death (19%, 5/27). One animal died due to septicemia caused by *Clostridium sordellii. Clostridium sordelli* has been described as cofactor in septicemic infections in cetaceans (44) and is known to cause fatal disease in humans (45). Trauma was suspected to cause the death of two WBDs from the Netherlands, although infectious diseases might have played a role in both cases. Intestinal volvulus was diagnosed in one animal from Schleswig-Holstein and considered as the cause of death in this individual. As the animal also showed ulceration of the stomach, gastrointestinal disease as a risk factor for the development of volvulus cannot be excluded in this case. Other possible risks linked to the development of volvulus, such as parasitic infection, bycatch or encephalitis causing changes in movement and behavior (46) were not observed in this animal. Among the animals from Germany, other diseases associated with spontaneous death include pneumonia, endoparasitosis, encephalitis and splenitis combined with hepatitis.

Suffocation as a consequence of bycatch frequently causes death and is considered as a threat to several cetacean species in the North Sea and North Atlantic (32, 47, 48) External scars or wounds indicating trauma or bycatch were not detected in any of the animals in this study except for one WBD (LA11) from the Netherlands, with possible trauma to the head and multifocal wounds and scarring on body and tailstock. Nevertheless, possible bycatch or entanglement in fishing gear prior to stranding cannot be ruled out, as external lesions can be missing. Lung edema has been linked to suffocation before, and was observed in other studies from Europe, dealing with post-mortem pathological findings (32, 49, 50), but was not significantly associated with bycatch in cetaceans from the Atlantic coast in a study dealing with discrimination of bycatch and other causes of death in ceataceans and pinnipeds (51), where multi-organ congestion, disseminated gas bubbles and red or bulging eyes were some of the lesions most commonly seen. Lung edema as an unspecific sign for bycatch was seen in nine animals from Germany and six animals from the Netherlands. As other signs were missing, it seems unlikely, that bycatch was a cause of death in any of the dolphins, which were examined in this study.

Bronchopneumonia was a common pathological finding in WBDs and AWSDs. In most cases the pneumonia seemed to be of bacterial origin, with *Streptococcus* spp. being the main detected microorganisms in affected animals. Beta-hemolytic streptococci have been associated with pneumonia, abscessation and septicemia in harbor porpoises (32, 52) and are known to cause pneumonic lesions in pinnipeds as well (53). The lungworms found in this study likely belonged to the Pseudaliidae family (Metastrongylidae), which typically infect odontocete respiratory tracts (23, 54). Interestingly, none of the cases with lungworm infections (7%, 2/27) showed signs of inflammation in this study. In comparison to other cetaceans and marine mammals inhabiting the North Sea, this is very unusual. Parasitic lung infections in harbor porpoises are common and often cause pneumonia, which can be severe and is frequently considered as the main cause of disease and death (23, 32, 34). The two cases in this study with nematode infections in the lung did not present any lesions. *Halocercus delphini*, a lung parasite infecting the common dolphin *(Delphinus delphis*) has been described to have no significant effect on their host (23). Little impact on the host could be possible for parasitic lung infections in WBDs as well, however more research is needed to substantiate this. Summarizing all organs, at least seven species of parasites from six families and four classes (Table 8) were found in the investigated dolphins showing a diverse parasite fauna, but not having any serious impacts on the health status of the investigated animals. Little information about parasitism and its impact in WBDs and AWSDs is available (23), warranting more research and systematic surveys.

**Table 8:** Parasite species and infection site in WBDs and AWSDs.

**Parasites**	**Distribution**
**NEMATODA**	
**Anisakidae**	
*Anisakis* sp.	Esophagus, stomach, intestine
*Pseudoterranova decipiens*	Stomach
**Crassicaudidae**	
*Crassicauda* sp.	Sub cutis, fascia, vas deferens
**Pseudaliidae**	
*Stenurus globicephalae*	Tympanic bullae, eustachian tube
**ACANTHOCEPHALA**	
**Polymorphidae**	
*Bolbosoma capitatum*	Intestine
**CESTODA**	
**Phyllobothriidae**	
*Phyllobothrium delphini*	Blubber
*Monorygma grimaldii*	Peritoneum
**TREMATODA**	
**Heterophyidae**	
*Pholeter gastrophilus*	Stomach, ampulla duodeni

Two animals from Schleswig-Holstein showed bronchitis and encephalitis and therefore displayed the typical general findings associated with morbillivirus infection in cetaceans (55). Morbillivirus infection has been linked to mass stranding events and fatal disease of cetacean species (55, 56), but in a different study dealing with morbillivirus in WBD, the virulence of the virus for WBDs was rated as uncertain (57). Virus antigens were detected in one WBD from Germany in this study. Although the gross and histological examination showed lesions in lung and brain, the virus was only detected in brain tissue. As the animal showed the typical pathological findings, it is likely, that the virus caused disease in this animal. The infected dolphin stranded alive and was euthanized, so if the virus was the cause of the stranding or if death of the animal would have occurred otherwise, remains unclear, but it might have played a role in emaciation and weakness.

Thymic cysts were a rare finding in this study, which seems striking, as it was more commonly found in other studies on thymic cysts in cetaceans (50, 58). As mentioned before, no definite statement on the occurrence of microcysts can be done, due to the lack of histological examinations. Concerning the age of the animals (two adults, one subadult) with macrocysts, the findings fit into the results of former studies, in which the development of thymic cysts in harbor porpoises and bottlenose dolphins (*Tursiops truncatus*) correlates with age and physiological involution of the thymus (58, 59).

The occurrence of ankylosis of the vertebrae, as observed in a mature, female WBD (WBD15) has been described in previous studies on *L. albirostris* and other cetaceans (26, 60). It seems to be a common finding in older WBD specimen, as sign of advanced spondylosis deformans, especially affecting females (26), although it can occur in younger animals, according to a study on harbor porpoises (60). WBD15 from this study was 5 years by growth layers in the dentine, and therefore still quite young. Another possibility apart from age related physical stress on the vertebral column can be an inflammatory reaction. Bacterial infections of the intervertebral disc or bacterial osteomyelitis might also lead to new bone formation, especially in younger animals (26). No signs of inflammation were described in WBD 15, but a large scar (15 × 2 cm) on the right side of the back, just ventral of the dorsal fin could be a sign for an older trauma, which primarily caused the reaction of the bone.

Here we present a broad overview of pathological findings and appearance of diseases in stranded white-beaked dolphins as well as three Atlantic white-sided dolphins in the North Sea. Data has been published after mass stranding events of WBDs in Denmark (21) and AWSDs in Ireland (15), but knowledge of disease pathology of both species is still scarce. Further studies and homogeneously collected data on pathology is needed to gain a deeper insight into diseases and evaluate their role for the species health and management and in potential future stranding events.

Strandings, abundance and pathological findings should be observed further in the years ahead, to be able to assess the health and population status of *Lagenorhynchus* species. Although WBDs and AWSDs are both listed as ‘least concern’ by the IUCN Red list, population trends for both species are still unknown. The potential impact of direct takes in certain parts of the Atlantic, as well as bycatch and combined anthropogenic threats, including those associated with climate change, need further assessment (18, 19, 61).

## Data Availability Statement

The datasets generated for this study are available on request to the corresponding author.

## Ethics Statement

Ethical review and approval was not required for the animal study because the animals from the Netherlands described in this study were not used for scientific or commercial testing. All were free-living dolphins which died in their natural environment and washed ashore, or live stranded and subsequently died of natural causes. None of the animals were euthanized. No consent from an Animal Use Committee is required when dealing with animals that died due to natural causes, as was the case here. Consequently, animal ethics committee approval was not applicable to this work. Specimens from Germany were collected within the German stranding network, which conducts work (collect and hold carcasses and samples from European protected species) on German strandings following appropriate licenses from the relevant authorities (Ministry of Energy, Agriculture, the Environment, Nature and Digitalization, Ministry of Agriculture, Environment and Rural areas, Permit Nr. DE 03 201 0046 21).

## Author Contributions

LS, MG, and US prepared the manuscript. LI, US, AG, MK, PW, and WB conducted necropsies, histological, immunohistochemical and virological examination. CE and EP-B performed the microbiological analyses. Parasitological investigations were done by JL and KL. All authors provided comments on the manuscript and approved the final version.

## Conflict of Interest

The authors declare that the research was conducted in the absence of any commercial or financial relationships that could be construed as a potential conflict of interest.

## References

[1] Kinze CC White-beaked dolphin: *Lagenorhynchus albirostris*. In: Perrin WF Wursig B Thewissen JGM editors. Encyclopedia of Marine Mammals. San Diego, CA: Academic Press (2009). p. 1255–8. 10.1016/B978-0-12-373553-9.00285-6

[2] Canning SJ Santos MB Reid RJ Evans PG Sabin RC Bailey N Seasonal distribution of white-beaked dolphins (*Lagenorhynchus Albirostris*) in UK waters with new information on diet and habitat use. J Marine Biol Assoc UK. (2008) 88:1159–66. 10.1017/S0025315408000076

[3] Galatius A Jansen OE Kinze CC Parameters of growth and reproduction of white-beaked dolphins (*Lagenorhynchus Albirostris*) from the North Sea. Mar Mamm Sci. (2013) 29:348–55. 10.1111/j.1748-7692.2012.00568.x

[4] Shirihai H Jarrett B Weißschnauzendelfin. In: Shirihai H Jarrett B, editors. Meeressäuger. Berlin: Kosmos (2008). p. 199–200.

[5] Jansen OE Leopold MF Meesters EH Smeenk C Are white-beaked dolphins *Lagenorhynchus Albirostris* food specialists? their diet in the southern North Sea. J Marine Biol Assoc UK. (2010) 90:1501–8. 10.1017/S0025315410001190

[6] Reijnders PJH Lankester K Status of marine mammals in the North Sea. Neth J Sea Res. (1990) 26:427–35. 10.1016/0077-7579(90)90098-2

[7] Northridge SP Tasker ML Webb A Williams JM Distribution and relative abundance of harbour porpoises (*Phocoena phocoena* L.), white-beaked dolphins (*Lagenorhynchus albirostris* Gray), and minke whales (*Balaenoptera acutorostrata* Lacepède) around the British Isles. ICES J Marine Sci. (1995) 52:55–66. 10.1016/1054-3139(95)80015-8

[8] Hammond PS Berggren P Benke H Borchers DL Collet A Heide-Jørgensen MP Abundance of harbour porpoise and other cetaceans in the North Sea and adjacent waters. J Appl Ecol. (2002) 39:361–76. 10.1046/j.1365-2664.2002.00713.x

[9] Hammond PS Lacey C Gilles A Viquerat S Boerjesson P Herr H Estimates of Cetacean Abundance in European Atlantic Waters in Summer 2016 From the SCANS-III Aerial and Shipboard Surveys. (2017). *Wageningen Marine Research*.

[10] Hammond PS Macleod K Berggren P Borchers DL Burt L Cañadas A Cetacean abundance and distribution in European Atlantic shelf waters to inform conservation and management. Biol Conserv. (2013) 164:107–22. 10.1016/j.biocon.2013.04.010

[11] Weir CR Stockin KA Pierce GJ Spatial and temporal trends in the distribution of harbour porpoises, white-beaked dolphins and minke whales off Aberdeenshire (UK), north-western North Sea. J Marine Biol Assoc UK. (2007) 87:327–38. 10.1017/S0025315407052721

[12] IJsseldijk LL Brownlow A Davison N Deaville R Haelters J Keijl G Spatiotemporal analysis in white-beaked dolphin strandings along the North Sea coast from 1991-2017. Lutra. (2018) 61:153–63.

[13] Evans PG Anderwald P Baines ME UK Cetacean Status Review. Report to English Nature and Countryside Council for Wales, UK. Oxford: Seawatch Foundation (2003).

[14] Sergeant DE Aubin DJ Geraci JR Life history and Northwest Atlantic status of the Atlantic white-sided dolphin, *Lagenorhynchus Acutus*. Cetology. (1980) 37:1–12.

[15] Rogan E Baker JR Jepson PD Berrow S Kiely O. A mass stranding of white-sided dolphins (*Lagenorhynchus Acutus*) in Ireland: biological and pathological studies. J Zool. (1997) 242:217–27. 10.1111/j.1469-7998.1997.tb05798.x21059883

[16] Weinrich MT Belt CR Morin D Behavior and ecology of the Atlantic white-sided dolphin (*Lagenorhynchus Acutus*) in coastal New England waters. Mar Mamm Sci. (2001) 17:231–48. 10.1111/j.1748-7692.2001.tb01268.x

[17] Banguera-Hinestroza E Evans PGH Mirimin L Reid RJ Mikkelsen B Couperus AS Phylogeography and population dynamics of the white-sided dolphin (*Lagenorhynchus Acutus*) in the North Atlantic. Conserv Genet. (2014) 15:789–802. 10.1007/s10592-014-0578-z

[18] Kiszka J Braulik G Lagenorhynchus Albirostris. The IUCN Red List of Threatened Species 2018. (2018) e.T11142A50361346 10.2305/IUCN.UK.20182.RLTS.T11142A50361346.en

[19] Braulik G (2019). Lagenorhynchus acutus. The IUCN Red List of Threatened Species 2019: e.T11141A50361160 10.2305/IUCN.UK.2019-3.RLTS.T11141A50361160.en

[20] Siebert U Müller S Gilles A Sundermeyer J Narberhaus I Kapitel VII, Artensteckbriefe marine säugetiere: Weißschnauzendelfin. In: Narberhaus I Krause J Bernitt U, editors. Bedrohte Biodiversität in der deutschen Nord- und Ostsee, Empfindlichkeiten gegenüber anthropogenen Nutzungen und den Effekten des Klimawandels. Bonn-Bad Godesberg: Bundesamt für Naturschutz (2012). p. 502–9.

[21] Alstrup AKO Jensen LF Hansen MS Kinze CC Jensen TH Necropsy findings of 11 white-beaked dolphins (*Lagenorhynchus Albirostris*) stranded in Denmark during 2008-2014. Aquat Mammals. (2016) 42:292–9. 10.1578/AM.42.3.2016.292

[22] Wohlsein P Puff C Kreutzer M Siebert U Baumgärtner W. Distemper in a dolphin. Emerg Infect Dis. (2007) 13:1959–61. 10.3201/eid1312.070309 18258062 PMC2876748

[23] Gibson DI Harris EA Bray RA Jepson PD Kuiken T Baker JR A survey of the helminth parasites of cetaceans stranded on the coast of England and wales during the period 1990-1994. J Zool. (1998) 244:563–74. 10.1111/j.1469-7998.1998.tb00061.x

[24] Galatius A Sonne C Kinze C Dietz R Jensen JEB. Occurrence of vertebral osteophytosis in a museum sample of white-beaked dolphins (*Lagenorhynchus Albirostris*) from Danish waters. J Wildl Dis. (2009) 45:19–28. 10.7589/0090-3558-45.1.19 19204332

[25] Kompanje EJO Differences between spondylo-osteomyelitis and spondylosis deformans in small odontocetes based on museum material. Aquat Mammals. (1995) 21:199–204.

[26] Kompanje EJO On the occurrence of spondylosis deformans in white-beaked dolphins *Lagenorhynchus Albirostris* (Gray, 1846) stranded on the Dutch coast. Zool Meded. (1995) 69:231–50.

[27] Osterhaus ADME Broeders HWJ Teppema JS Kuiken T House JA Vos HW. Isolation of a virus with rhabdovirus morphology from a white-beaked dolphin (*Lagenorhynchus Albirostris*). Arch Virol. (1993) 133:189–93. 10.1007/BF01309754 8240009

[28] Bertulli CG Cecchetti A Van Bressem MF Van Waerebeek K Skin disorders in common minke whales and white-beaked dolphins off Iceland, a photographic assessment. Skin. (2012) 5:29–40.

[29] Buck JD Spotte S Microbiology of captive white-beaked dolphins (*Lagenorhynchus Albirostris*) with comments on epizootics. Zoo Biol. (1986) 5:321–9. 10.1002/zoo.1430050402

[30] Keijl GO Begeman L Hiemstra S IJsseldijk LL Kamminga P Cetaceans stranded in the Netherlands in 2008-2014. Lutra. (2016) 59:75–107.

[31] Lockyer C A review of factors involved in zonation in odontocete teeth, and an investigation of the likely impact of environmental factors and major life events on harbour porpoise tooth structure. Rep Int Whal Comm Spec Issues. (1995) 16:511–30.

[32] Siebert U Wünschmann A Weiss R Frank H Benke H Frese K. Post-mortem findings in harbour porpoises (*Phocoena Phocoena*) from the German North and Baltic Seas. J Comp Pathol. (2001) 124:102–14. 10.1053/jcpa.2000.0436 11222006

[33] IJsseldijk LL Brownlow AC Mazzariol S Best practice on cetacean post mortem investigation and tissue sampling. Joint ASCOBANS/ACCOBAMS Doc. (2019) 1–73. 10.31219/osf.io/zh4ra

[34] Lehnert K Raga JA Siebert U. Macroparasites in stranded and bycaught harbour porpoises from German and Norwegian waters. Dis Aquat Org. (2005) 64:265–9. 10.3354/dao064265 15997825

[35] Arnold PW Gaskin DE. Lungworms (*Metastrongyloidea: Pseudaliidae*) of harbor porpoise *Phocoena Phocoena* (L. 1758). Can J Zool. (1975) 53:713–35. 10.1139/z75-087 1139462

[36] Delyamure SL Gel'Mintofauna Morskikh Mlekopitayushchikh v Svete Ikh Ekologii I Filogenii (Helminthofauna of Marine Mammals; Ecology And Phylogeny). Moscow, USSR: Academy of Science, Laboratory for Helminthology *Translated from Russian: Jerusalem, Israel: Israel Program for Scientific Translations*, 1968. (1955).

[37] Siebert U Rademaker M Ulrich SA Wohlsein P Ronnenberg K Prenger-Berninghoff E. Bacterial microbiota in harbor seals (*Phoca Vitulina*) from the North Sea of schleswig-holstein, Germany, around the time of morbillivirus and influenza epidemics. J Wildl Dis. (2017) 53:201–14. 10.7589/2015-11-320 28139956

[38] Bisping W Amtsberg G Colour atlas for the diagnosis of bacterial pathogens in animals. (1988). Berlin: Paul Parey Scientific Publishers.

[39] Burkhardt F. Mikrobiologische Diagnostik. Stuttgart: G. Thieme Verlag. (1992).

[40] Carter GR Chengappa MM Roberts AW. Essentials of Veterinary Microbiology. 5th ed. Baltimore: Williams & Wilkins. (1995).

[41] Harder TC Moennig V Greiser-Wilke I Barrett T Liess B. Analysis of antigenic differences between sixteen phocine distemper virus isolates and other morbilliviruses. Arch Virol. (1991) 118:261–8. 10.1007/BF01314036 2069507

[42] Baumgärtner W Boyce RW Weisbrode SE Alldinger S Axthelm MK Krakowka S. Histologic and immunocytochemical characterization of canine distemper-associated metaphyseal bone lesions in young dogs following experimental infection. Vet Pathol. (1995) 32:702–9. 10.1177/030098589503200612 8592806

[43] Key M Immunohistochemical staining methods, In: Kumar GL Rudbeck L, editors. Immunohisto- Chemical Staining Methods. 5th edition Carpinteria, CA: Dako Corporation (2009). p. 57–60.

[44] Suárez-Santana CM Sierra E Díaz-Delgado J Zucca D de Quirós YB Puig-Lozano R. Prostatic lesions in odontocete cetaceans. Vet Pathol. (2018) 55:466–72. 10.1177/0300985818755252 29402205

[45] Aldape MJ Bryant AE Stevens DL. *Clostridium sordellii* infection: epidemiology, clinical findings, and current perspectives on diagnosis and treatment. Clin Infect Dis. (2006) 43:1436–46. 10.1086/508866 17083018

[46] Begeman L Leger JS Blyde DJ Jauniaux TP Lair S Lovewell G. Intestinal volvulus in cetaceans. Vet Pathol. (2013) 50:590–6. 10.1177/0300985812465327 23150643

[47] Couperus AS Interactions between Dutch midwater-trawl and Atlantic white-sided dolphins (*Lagenorhynchus acutus*) southwest of Ireland. J Northwest Atl Fish Sci. (1997) 22:209–18. 10.2960/J.v22.a16

[48] Mannocci L Dabin W Augeraud-Véron E Dupuy JF Barbraud C Ridoux V. Assessing the impact of bycatch on dolphin populations: the case of the common dolphin in the eastern North Atlantic. PLoS ONE. (2012) 7:e32615. 10.1371/journal.pone.0032615 22393423 PMC3290591

[49] Kuiken T Simpson VR Allchin CR Bennett PM Codd GA Harris EA. Mass mortality of common dolphins (*Delphinus Delphis*) in south west England due to incidental capture in fishing gear. Vet Rec. (1994) 134:81–9. 10.1136/vr.134.4.81 8178416

[50] Wunschmann A Siebert U Frese K. Thymic cysts in harbor porpoises (*Phocoena phocoena*) from the German North Sea, Baltic Sea, and waters of Greenland. Vet Pathol. (1999) 36:391–6. 10.1354/vp.36-5-391 10490206

[51] de Quirós YB Hartwick M Rotstein DS Garner MM Bogomolni A Greer W. Discrimination between bycatch and other causes of cetacean and pinniped stranding. Dis Aquat Org. (2018) 127:83–95. 10.3354/dao03189 29384478

[52] Swenshon M LaÈmmler CH Siebert U. Identification and molecular characterization of beta-hemolytic streptococci isolated from harbor porpoises (*Phocoena phocoena*) of the North and Baltic Seas. J Clin Microbiol. (1998) 36:1902–6. 10.1128/JCM.36.7.1902-1906.1998 9650933 PMC104949

[53] Higgins R. Bacteria and fungi of marine mammals: a review. Can Vet J. (2000) 41:105–16. 10723596 PMC1476275

[54] Lehnert K Seibel H Hasselmeier I Wohlsein P Iversen M Nielsen NH Increase in parasite burden and associated pathology in harbour porpoises (*Phocoena Phocoena*) in West Greenland. Polar Biol. (2014) 37:321–31. 10.1007/s00300-013-1433-2

[55] Kennedy SEAMUS. Morbillivirus infections in aquatic mammals. J Comp Pathol. (1998) 119:201–25. 10.1016/S0021-9975(98)80045-5 9807724

[56] Raga JA Banyard A Domingo M Corteyn M Van Bressem MF Fernández M. Dolphin morbillivirus epizootic resurgence, Mediterranean Sea. Emerging Infect Dis. (2008) 14:471–3. 10.3201/eid1403.071230 18325265 PMC2570809

[57] Van Elk CE Van de Bildt MWG Jauniaux T Hiemstra S Van Run PRWA Foster G. Is dolphin morbillivirus virulent for white-beaked dolphins (*Lagenorhynchus Albirostris*)? Vet Pathol. (2014) 51:1174–82. 10.1177/0300985813516643 24399208

[58] Cowan DF. Involution and cystic transformation of the thymus in the bottlenose dolphin, *Tursiops Truncatus*. Vet Pathol. (1994) 31:648–53. 10.1177/030098589403100603 7863579

[59] Yap X Deaville R Perkins MW Penrose R Law RJ Jepson PD. Investigating links between polychlorinated biphenyl (PCB) exposure and thymic involution and thymic cysts in harbour porpoises (*Phocoena Phocoena*). Mar Pollut Bull. (2012) 64:2168–76. 10.1016/j.marpolbul.2012.07.038 22917837

[60] Kinze C C Note on the occurrence of spondylitis deformans in a sample of harbour orpoises (*Phocoena phocoena* (L.)) taken in danish waters. Aquat Mammals. (1986) 12:25–27.

[61] Lambert E Pierce GJ Hall K Brereton T Dunn TE Wall D. Cetacean range and climate in the eastern North Atlantic: future predictions and implications for conservation. Glob Chang Biol. (2014) 20:1782–93. 10.1111/gcb.12560 24677422

